# Regulation of macrophage activation by lactylation in lung disease

**DOI:** 10.3389/fimmu.2024.1427739

**Published:** 2024-07-04

**Authors:** Yungeng Wei, Hua Guo, Shixing Chen, Xiao Xiao Tang

**Affiliations:** ^1^ State Key Laboratory of Respiratory Disease, National Clinical Research Center for Respiratory Disease, National Center for Respiratory Medicine, Guangzhou Institute of Respiratory Health, The First Affiliated Hospital of Guangzhou Medical University, Guangzhou, China; ^2^ Guangzhou Laboratory, Bio-island, Guangzhou, China

**Keywords:** macrophage, lactylation, immune regulation, metabolism, lung disease

## Abstract

Lactylation is a process where lactate, a cellular metabolism byproduct, is added to proteins, altering their functions. In the realm of macrophage activation, lactylation impacts inflammatory response and immune regulation. Understanding the effects of lactylation on macrophage activation is vital in lung diseases, as abnormal activation and function are pivotal in conditions like pneumonia, pulmonary fibrosis, COPD, and lung cancer. This review explores the concept of lactylation, its regulation of macrophage activation, and recent research progress in lung diseases. It offers new insights into lung disease pathogenesis and potential therapeutic targets.

## 1 Introduction

Lung diseases, a group of disorders that affect the respiratory system, are caused by a variety of factors, including genetic predisposition, environmental factors, infections, lifestyle, and occupational exposures (1). There is a wide range of lung diseases including, but not limited to, asthma, chronic obstructive pulmonary disease, pneumonia, tuberculosis, pulmonary fibrosis and lung cancer (1–4). The symptoms and severity of these diseases range from mild to severe, affecting respiratory function and life quality of the patients. The pathogenesis of lung diseases is complex, involving inflammatory response, cellular damage, tissue repair and fibrosis. Identification of pathogenic mechanisms and novel therapeutic approaches is particularly important to reduce the disease burden.

Epigenetic modifications are chemical alterations to DNA and proteins, rather than changes to the gene sequence itself. These changes mainly encompass DNA methylation, post-translational modifications (PTM) of histones, and non-coding RNAs. They have the potential to influence gene expression and the transmission of genetic information. Epigenetic modifications are indispensable in cell differentiation, disease progression, and environmental adaptation, playing a vital role in the development and survival of organisms. A strong correlation exists between cellular metabolism and epigenetic modifications (5). Metabolites produced during metabolic processes can impact epigenetic alterations in cells, which in turn regulate metabolic programming. The interaction between the two processes collaboratively modulates gene expression and cellular function (6).

Lactate in moderate amounts is not harmful to the body as it is a normal metabolic byproduct that can be efficiently eliminated under aerobic conditions. However, its excessive accumulation can disrupt the body’s homeostasis. Accumulated lactate triggers cell signaling pathways that regulate inflammatory progression and tumor immune tolerance. It is involved in various diseases and inhibits acute inflammation (7, 8). The tissue damage repair process is regulated by cytokines and growth factors. Lactate acts as an energy substrate to meet the high metabolic demands of repair (9, 10). Lactate and lactate-mediated activation play a significant role in various aspects of tumor progression, such as cell proliferation, invasion, angiogenesis, immune tolerance, and immune cell evasion (11, 12). Lactate was once seen as a byproduct of anaerobic metabolic processes, accumulating in the tissue microenvironment of inflammatory and neoplastic diseases, whereas some studies take lactate as a signaling molecule (6). Histone lactylation, a post-translational modification first described in 2019, involves glycolysis-derived lactate acting as a substrate for histone lactylation. This process directly influences the transcription of chromatin genes by modifying histone lysine residues (13). Further studies show that histone lactylation plays a role in regulating macrophage M1/2 polarization (14), cellular reprogramming (15), and tumorigenesis (16, 17). As research continues to deepen and expand, it has been found that lactate-driven lactylation occurs not only in histones, but also in other organelles and non-histone proteins (17, 18). This implies the important role of lactylation in various physiological and pathological processes. Lactate is now recognized beyond its metabolic function, as lactylation has emerged as a new area of research in protein post-translational modifications, particularly in the realms of tumor biology and immunity.

Macrophages, the firstline of immune response, uphold tissue homeostasis by recognizing and eliminating pathogens, killing target cells, presenting antigens, and regulating immunity. However, their excessive aggregation and activation can also cause tissue damage (1, 19, 20). Macrophages react to microorganisms and harmful factors by initiating inflammatory response to combat pathogenic threats; the shift from a pro-inflammatory to a reparative state is crucial for anti-inflammatory effects and restoring homeostasis. The molecular mechanisms governing this transition remain unclear. Lactylation regulates macrophage activation and function through diverse pathways. Further research on lactylation in macrophages could enhance our understanding of macrophage function and immune regulation, as well as offer new insights and targets for treating related diseases.

## 2 Lactate and lactylation

### 2.1 Sources of lactate

**Figure 1:**
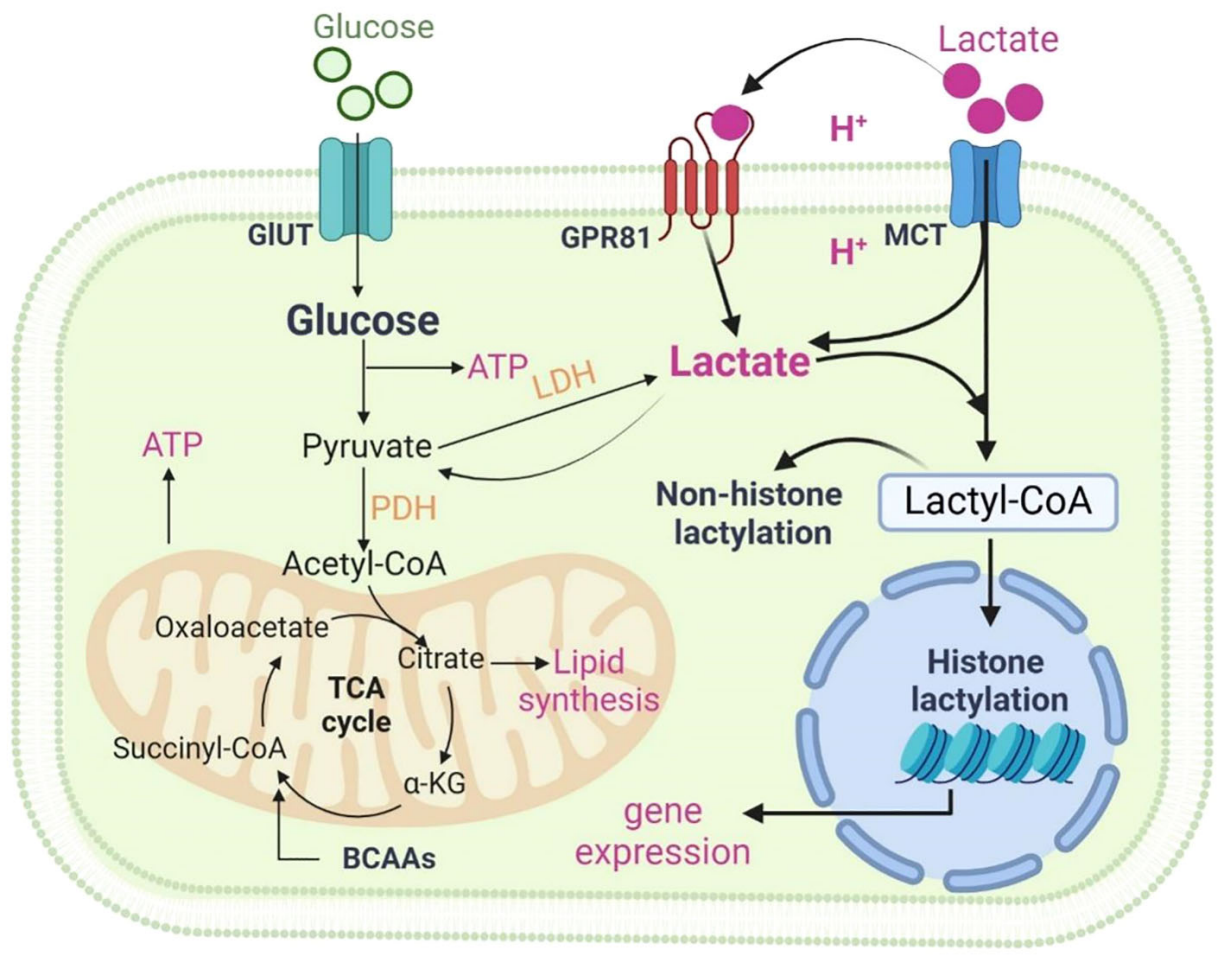
Metabolism of lactate. Two pathways of metabolic breakdown of lactic acid: lactate can be converted to pyruvate entering the tricarboxylic acid (TCA) cycle, or it can generate lactyl-coenzyme A (Lactyl-CoA), which is involved in lactylation of histones and non-histone proteins. Lactate transfers inter- and intracellularly through MCTs and GPR81. Figure was created with BioRender.com.

Lactate, an organic acid with the chemical formula C_3_H_6_O_3_, is a hydroxy acid and is also known as hydroxypropionic acid due to the hydroxyl group (-OH) in its chemical structure (21). Lactate is typically produced in three isomeric forms: D-lactate, L- lactate, and racemic mixture DL-lactate (22). L-lactate is the primary form in mammals, while D-lactate is an atypical mammalian metabolite and is primarily produced by glyoxalase to metabolize methylglyoxal (23). Lactate plays a crucial metabolic role in organisms via the family of transporter proteins monocarboxylic acid transporters (MCTs) (24) and its receptor G-protein-coupled receptor (GPR) 81 (25, 26). Lactate transfers inter- and intracellularly through MCTs and GPR81 to regulate physiological and pathological processes (**Figure 1**) (23). In mammalian cells, lactate is mainly produced from pyruvate, which is converted to lactate by lactate dehydrogenase A (LDHA). Intracellular pyruvate is derived from glycolysis and glutamine metabolism (27).

The metabolic breakdown of lactate involves two pathways: lactate can be converted to pyruvate to enter the tricarboxylic acid (TCA) cycle, or it can generate lactyl-coenzyme A (Lactyl-CoA), which is involved in lactylation of histones and non-histone proteins. Lactate metabolism involves the action of several key enzymes. Some of the major ones include: Lactate dehydrogenase (LDH), which converts pyruvate to lactate; Pyruvate kinase, facilitating the reaction between pyruvate and ADP to form pyruvate and ATP; Pyruvate dehydrogenase (PDH), converting pyruvate to Lactyl-CoA.

In addition to intracellular production, lactate can enter target cells through non-channel pathways or MCTs-mediated intercellular shuttling, influencing cellular physiological and pathological processes (24). Lactate shuttling involves the transportation and transformation of lactate within and between cells. This process allows rapid movement of lactate from its production site to other tissues or cells for further metabolism. Essential for maintaining lactate balance, shuttling regulates the concentration of lactate both inside and outside cells. MCT proteins primarily mediate lactate shuttling; MCT1 exhibits high lactate affinity, facilitating bidirectional transport across cell membranes, while MCT4, prevalent in highly glycolytic tissues, acts as a unidirectional transporter primarily for lactate export (28).

Factors influencing lactate levels include: 1. lactate production rate, determined by glycolysis and hypoxia levels. 2. lactate transport and metabolism, crucial for maintaining intracellular and extracellular lactate balance. 3. lactate clearance, involving liver and tissue processes that regulate its levels.

### 2.2 Lactylation

Lactylation, a histone modification introduced in 2019, has shown significant potential. Histone lysine lactylation has been observed to accumulate at gene promoters in cells under hypoxia, interferon (IFN)-γ, lipopolysaccharide (LPS), or bacterial attack, directly influencing gene expression for regulating bacterially-infected M1 macrophages (13, 16). As we mentioned earlier, lactate contains several forms and it has been found that L-lactate, not D-lactate, is used in the histone lactylation process (29). Apart from histones, other non-histone proteins can also undergo lactylation. Non-histone protein modifications are crucial in various cellular processes including gene transcription, DNA damage repair, cell division, signal transduction, protein folding, autophagy, and metabolism (30). Initial study on lactylation focused on macrophages, the cell type where histone lactylation was discovered, and their related diseases. To date, research on lactylation has focused on a diverse array of cell types (30). Remarkably, lactylation has been detected in a wide range of proteins found in the nucleus, cytoplasm, mitochondria, endoplasmic reticulum, and cell membrane (17, 18), suggesting that protein lactylation may be involved in regulating various biological activities. These findings emphasize the significant influence of lactylation as a posttranslational modification and suggest its potential role in a variety of cellular processes.

Histone lactylation by “writers” and “erasers”: In the process of histone modification, “writers” and “erasers” play significant roles in regulating gene expression dynamically and reversibly. In histone lactylation, the “writers” transfers lactate-generated lactyl-coenzyme A to the histone lysine tail for lactylation. The most studied lactylation writers are P300 (acetyltransferase), and two other histone acetyltransferase (HAT) family proteins, males absent on the first (MOF), and general control non-depressible 5 (GCN5) (13, 31, 32). The latest study reveals that lysine acetyltransferase 7 (HBO1) serves as a lactyltransferase to mediate a histone Kla (33). Other proteins such as CREBBP (CBP), peptidyl-lysine N-acetyltransferase YiaC, and lysine acetyltransferase 8 (KAT8) may also act as potential regulators of lactylation (30, 34). On the contrary, the “erasers” like histone deacetylase (HDAC)1–3 and Sirtuins (SIRT)1–3 are responsible for de-lactylation (29, 31, 32). Nevertheless, despite these discoveries, the specific enzymes and mechanisms that play a role in regulating lactylation in cells are still elusive, underscoring the necessity for additional research in this field.

### 2.3 The role of lactylation in pathogenic mechanisms

Lactylation is an important post-translational modification that can alter protein function and stability. It may influence organism function in various ways. Lactylation can modify gene expression and protein function, thus impacting their activity and stability within the cell (18, 32, 35, 36). This alteration can result in disruptions in intracellular signaling pathways, thereby affecting normal cell function. Additionally, lactylation might influence protein interactions with other proteins or ligands (37, 38), thereby affecting intracellular protein signaling and metabolic regulation. Furthermore, lactylation could disrupt progression of normal intracellular metabolic pathways (39–41), leading to irregularities in energy and material metabolism, consequently affecting cell function.

In essence, abnormal lactylation could detrimentally impact physiological cell functions, contributing to disease onset and progression. Hence, investigating the pathogenic mechanism of lactate modification is critical for understanding disease development at a molecular level and devising relevant therapeutic approaches.

## 3 Regulation of macrophage activation by lactylation

### 3.1 Subtypes of lung macrophages and their types of activation

**Figure 2:**
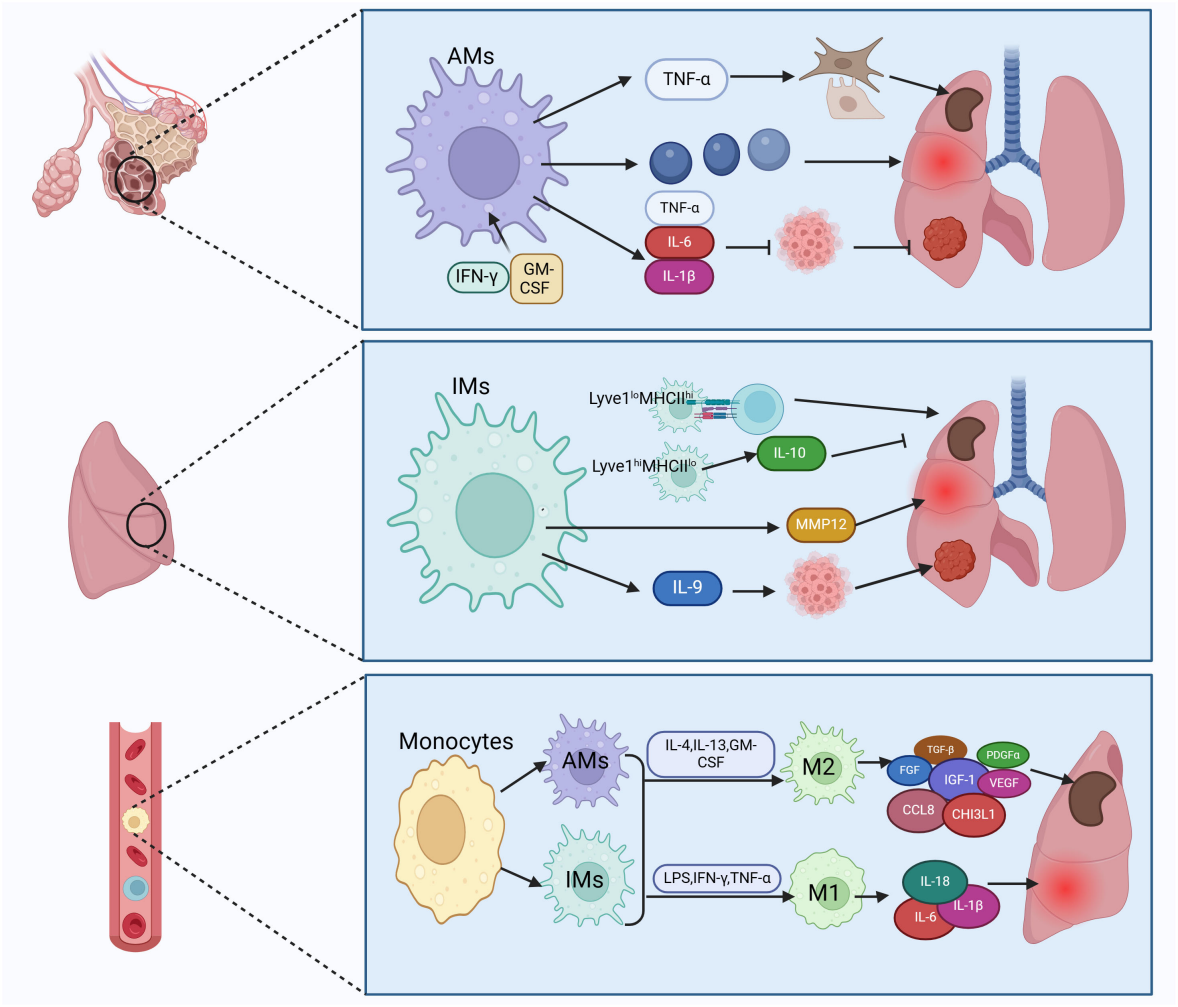
Subtypes of lung macrophages and their types of activation. Macrophages in lung tissue are categorized into tissue-resident macrophages and recruited mononuclear macrophages. Tissue-resident macrophages consist of alveolar macrophages (AMs) and interstitial macrophages (IMs). AMs can trigger fibroblasts and epithelium by secreting TNF-α, leading to fibrosis. They also release inflammatory factors causing lung injury and secrete IL-6 to inhibit lung cancer. IMs can enhance lung fibrosis, inflammation, and cancer by antigen presentation and secretion of IL-10, MMP12, and IL-9. Recruited monocytes transform into AMs and IMs, which can further differentiate into classically activated macrophages (M1) and alternatively activated macrophages (M2). M1 macrophages produce pro-inflammatory factors like IL-6 to boost lung inflammation, while M2 macrophages secrete TGF-β to induce pulmonary fibrosis. Figure was created with BioRender.com.

Two types of macrophages exist in human lung tissue: tissue-resident macrophages (TRMs) and recruited monocyte-derived macrophages (**Figure 2**). Among TRMs, two main subtypes are identified: alveolar macrophages (AMs) and interstitial macrophages (IMs) (1). AMs are located within the alveoli, characterized by surface markers CD11b^low^ CD11c^++^ CD169^+^, while IMs reside in the lung parenchyma with surface markers CD11b^+^ CD11c^low^ CD169^-^ (4, 42, 43).

AMs are the predominant macrophages in the lungs and play a vital role in maintaining lung homeostasis, defending against pathogens, regulating lung inflammation, and possessing self-renewal capabilities (42, 44). AMs may promote fibrosis. Lineage tracing experiments in murine fibrosis models have shown an expansion of the AMs pool derived from monocytes expressing genes that promote fibrosis, and removing this subset of macrophages has been found to alleviate fibrosis (45, 46). During acute lung injury, AMs coordinate the initiation and resolution of inflammation to restore internal balance (47). In cases of chronic lung inflammation, AMs cause lung damage by producing various pro-inflammatory mediators such as chemokines, cytokines, proteases, reactive oxygen intermediates (ROI), and reactive nitrogen intermediates (RNI). Research suggests a link between matrix metalloproteinases (MMPs) and destruction or remodeling of lung tissue in chronic obstructive pulmonary disease (COPD) (2). Interestingly, TNF-α released by AMs promotes secretion of granulocyte-macrophage colony-stimulating factor (GM-CSF) from alveolar epithelial cells, supporting alveolar epithelial repair (48). The role of AMs in lung tumors is intricate (49). AMs obtained from lung cancer patients, when exposed to IFN-γ or GM-CSF, secrete TNF-α, IL-6, and IL-1β, enhancing anti-tumor activity (50). However, other studies indicate that many anti-tumor functions of AMs, such as phagocytosis and expression of cytokines that activate adaptive immunity, diminish as non-small cell lung cancer (NSCLC) progresses (51), suggesting a role of AMs in fostering an immunosuppressive microenvironment and immune dysfunction.

In contrast to the extensive research on AMs, there is relatively less study on IMs. IMs within tissue-resident macrophages can be further classified into Lyve1^lo^MHCII^hi^ and Lyve1^hi^MHCII^lo^ subtypes, each exhibiting distinct functional characteristics. Lyve1^lo^MHCII^hi^ IMs excel in antigen presentation, while Lyve1^hi^MHCII^lo^ IMs possess the ability to inhibit tissue fibrosis. Lyve1^hi^MHCII^lo^ IMs play an important role in suppressing collagen deposition, immune cell infiltration, and tissue inflammation, and their absence can exacerbate bleomycin-induced lung fibrosis (52). In COPD, IMs have been found to secrete matrix metalloproteinase 12 (MMP12), promoting formation of lung emphysema (53). In addition, IMs regulate virus-induced inflammation, serving as the primary source of the anti-inflammatory cytokine IL-10 in response to influenza A virus (IAV) infection and exposure to bacterial DNA (54). In the development of lung tumors, IMs act as the main tumor-associated macrophages (TAMs), with IL-9 signaling promoting the conversion of monocytes to IMs (19). Moreover, IMs represent the primary responders to IL-9 in lung cancer, and blocking IL-9 signaling on lung macrophages restricts the growth of murine lung tumors (55).

In the progression and advancement of pulmonary fibrosis, both AMs and IMs have the ability to polarize into classically activated macrophages (M1) and alternatively activated macrophages (M2), respectively (56, 57). Depending on the specific activating signals, M2 macrophages can be further classified into four subtypes: M2a, M2b, M2c, and M2d (20). In the early phases of lung damage, M1 macrophages get activated by LPS, IFN-γ, and TNF-α. These activated M1 macrophages contribute to pathogen elimination and escalate inflammation. With the progression of inflammation, the quantity of M2 macrophages rises as triggered by IL-4, IL-13, GM-CSF, and other factors, dominating the immune response. M2 macrophages, via the release of fibrosis-promoting molecules like TGF-β, fibroblast growth factor (FGF), platelet-derived growth factor alpha (PDGFα), insulin-like growth factor-1 (IGF-1), and vascular endothelial growth factor (VEGF), actively promote extracellular matrix (ECM) expansion and tissue restoration (58, 59). The prevalence of M2 macrophages tends to be elevated in idiopathic pulmonary fibrosis (IPF) (60). Macrophages stimulate fibroblasts by releasing fibrosis-promoting factors and remodeling ECM, thus advancing lung fibrosis (61–63). Studies have shown increased expression of the M2 macrophage-related chemokine CCL18 in bronchoalveolar lavage fluid (BALF) and serum of patients with IPF. The level of CCL18 is linked to the mortality rate of IPF patients (64, 65). CCL18 attracts T lymphocytes and maintains a positive feedback loop with lung fibroblasts to enhance collagen production (66). Another marker of M2 activation is chitinase 3-like protein 1 (CHI3L1), which is also elevated in the BALF of IPF patients and indicates a poor prognosis (4). Research indicates that inhibiting M2 macrophage polarization can reduce bleomycin-induced lung fibrosis in rats (67).

### 3.2 Regulation of macrophage M1/M2 by lactylation

Lactylation is a common post-translational modification that can influence protein stability, activity, and subcellular localization. Recent study indicates that lactylation can influence the M1/M2 polarization state of macrophages (14). As both AMs and IMs can undergo M1/M2 polarization, we focus on the regulatory mechanism of macrophage M1/M2 by lactylation. M1 macrophages typically trigger pro-inflammatory responses and cytotoxic activity, while M2 macrophages are linked to anti-inflammatory responses and reparative functions (56, 57). Macrophages polarize into different phenotypes in response to local microenvironmental cues. Activation of glycolytic pathways enhances production and release of lactate, leading to polarization of macrophages towards the M1 phenotype. In the early stages of inflammation, lactylation promotes M1 polarization of macrophages, with nitric oxide synthase 2 (NOS2) protein level peaking at 12 hours post-stimulation and decreasing thereafter (13). Furthermore, genes like arginase 1 (ARG1), modified by H3K18la, show significant upregulation primarily at 16–24 hours. The study demonstrates that the aerobic glycolysis observed during M1-type macrophage polarization sets off a “lactate clock” that drives M2-type gene expression during the later stages of M1 polarization by facilitating the lactylation of histone H3 lysine 18 (13). Mitochondria, the key metabolic organelles in cells, could adjust their energy production to support the transition of macrophages from a pro-inflammatory to a proresolving state by undergoing fission and fusion processes. Inflammatory M1 cells display elongated mitochondria, and blocking mitochondrial fusion enhances histone lactylation by affecting lactate levels, promoting M2 polarization in macrophages (68). Oncogenic M2-type macrophages isolated from melanoma and lung cancer tissues in mice exhibit a strong positive correlation with lactylation levels. This suggests that elevated lactate and histone lactylation in macrophages may play a role in regulating macrophage M2 polarization.

### 3.3 The role of lactylation in macrophage activation

Lactylation is a post-translational modification process that modifies proteins by adding lactate groups, altering their function and stability. Macrophages, vital in immune defense, primarily engage in phagocytosis and eliminating pathogens and waste. The interplay between lactylation and macrophage function is significant. Firstly, lactylation can modify macrophage activity and function by influencing protein structure and stability (18, 32, 35, 36), thus affecting cellular function. Lactate inhibits Toll-like receptor (TLR)-mediated monocyte-macrophage activation (26, 69), delays protein kinase B (Akt) phosphorylation and IκBα degradation. It also inhibits secretion of cytokines including tumor necrosis factor α (TNF-α), interleukin-23 (IL-23), and chemokines C-C motif chemokine ligand (CCL)2 and CCL7 (70). Lactylation plays a key role in regulating macrophage activity. Secondly, lactylation influences macrophage metabolic processes. The correlation between lactylation and macrophage metabolism is notable (71). The boost in VEGF production by polarized macrophages establishes a beneficial cycle that enhances angiogenesis and supports postischemic hemotransfusion and muscle regeneration (72). As a result of tissue hypoxia, lactate can also act as a marker for insufficient tissue perfusion. In clinical practice, monitoring serum lactate level is important for managing patients with ischemic injuries (73).

Macrophages require substantial energy and nutrients during phagocytosis and waste removal, with lactylation affecting their metabolic functions by controlling protein degradation and synthesis, energy metabolism, and other pathways (39–41). Lactylation can also influence metabolic functions of macrophages via altering their absorption and use of nutrients. Multiple connections between lactylation and other epigenetic changes influence macrophage activities and metabolic processes. Lactylation and methylation modifications frequently interact at the cellular level. A study verified the presence of H3K18la enrichment at the m^6^A reader YTH N6-methyladenosine RNA binding protein F2 (YTHDF2) promoter, demonstrating that H3K18la regulates YTHDF2 transcription (74). Hyper-H3K18la promotes the transcription of YTHDF1 in AECs of arsenite-related IPF (75), suggesting that lactylation adjustments can influence the methylation levels of DNA and histones, which in turn affect gene expression and transcriptional control. This interplay holds significant implications for the immune and inflammatory responses of macrophages. Lactylation and acetylation also collaborate within the nucleus. The levels of lactate and acetyl-CoA synergistically determine the cell fate and are manifested in histones as epigenetic marks (i.e., lactylation and acetylation) (76). In macrophages, the interplay between lactylation and acetylation can influence the inflammatory response and antigen presentation (17). Lactylation and ubiquitination also interact in the regulation of protein degradation and stability. Lactylation can control the protein degradation rate via altering the ubiquitination levels of proteins (3, 5, 77). This interaction plays an essential role in antigen processing and immunomodulation in macrophages. As previously stated, lactate-induced lactylation is integral to macrophage activation. However, the investigation into the crosstalk between lactylation and other epigenetic modifications, particularly in macrophages, is still in its nascent stages.

## 4 Lactylation modulates macrophage activation in lung disease

### 4.1 Lactylation regulates macrophage activation in inflammatory lung diseases

Recent research indicates that lactylation affects macrophage activation and progression of lung inflammation. Increased lactate was found in BALF from particulate matter (PM2.5)-exposed mice and lactylation was increased in macrophages (78). Lactylation has been shown to potentially stimulate M1-type macrophage activation, which intensifies inflammatory reactions. Activated macrophages release pro-inflammatory cytokines that worsen inflammation in the lungs. In sepsis patients, blood lactate levels correlate with high mobility group box 1 (HMGB1) levels, a type of damage-associated molecular pattern (DAMP), from activated macrophages. Macrophages in sepsis can induce HMGB1 lactylation by absorbing extracellular lactate through MCTs. Lactylated HMGB1 is then released by macrophages, disrupting endothelial integrity, increasing vascular permeability, and contributing to endothelial cell barrier dysfunction, thereby promoting sepsis progression (17). Pyroptosis is a programmed cell death associated with inflammatory diseases, occurring in macrophages mostly (79–81). In non-canonical pyroptosis, NEDD4 E3 ubiquitin protein ligase (NEDD4) acts as a negative regulator of Caspase-11, and lactylated NEDD4 leads to macrophage pyroptosis (82), suggesting that lactylation might enhance inflammation by influencing pyroptosis. Moreover, dexamethasone (DEX) treatment inhibits protein lactylation and pyroptosis in macrophages, thereby attenuating ovalbumin-induced aeroallergen (83). Interestingly, a study shows that lactate can inhibit M1 macrophage polarization and increase histone H3K18 lactylation in macrophages, thereby suppressing macrophage pyroptosis (84). Additionally, research indicates that lactylation can drive M2-type macrophage activation (14, 85), promoting anti-inflammatory responses and tissue repair. Drug delivery by nanoplatform-based system significantly decreased histone lactylation levels in LPS-induced macrophages and induced macrophage towards to M2 polarization, thereby attenuating LPS-induced acute lung injury in mice (86). Activated M2 macrophages release anti-inflammatory cytokines to dampen inflammation and support tissue healing. In spite of the controversy, these studies suggest that further investigation of the mechanisms by which lactylation regulates macrophage activation, as well as the development of drugs targeting lactylation, may be beneficial to control acute inflammation in lung tissues.

### 4.2 Effect of lactylation modulating macrophage activation on pulmonary fibrosis

Pulmonary fibrosis is a chronic progressive lung disease characterized by proliferation and deposition of fibrous tissue in the lung, leading to structural damage and impaired function (87). Abnormal activation and function of macrophages are closely tied to pulmonary fibrosis (55, 60, 88). When lung tissue is injured or inflamed, macrophages release inflammatory and growth factors, fueling the inflammatory response and fibrotic progression (55, 58). Moreover, macrophages stimulate fibrosis-related cell growth and collagen synthesis, contributing to lung scarring (89).

Upregulation of the TGF-β/Smad2 pathway is critical in fibrosis development and worsens fibrotic conditions. Lactylation of Snail1, a TGF-β transcription factor, enhances its nuclear localization and binding to the TGF-β promoter, leading to increased TGF-β production (90). Histone H3K18 lactylation interacts with m^6^A methylation to promote organic arsenic-induced pulmonary fibrosis (74). Also, lactate from the lung tissue can induce a pro-fibrotic phenotype in macrophages by promoting lactylation (85). This pro-fibrotic effect of lactylation may modulate macrophage metabolic phenotype and M2 polarization. During pulmonary fibrosis progression, glucose consumption increases, shifting macrophages’ metabolic status from lipid to glucose metabolism, particularly in the TCA cycle, and increasing glycolysis/gluconeogenesis in macrophages during the fibrotic phase (91, 92). Pyruvate kinase M2 (PKM2) plays a critical role in mediating metabolic adaptations in pro-inflammatory macrophages. Lactylation of PKM2 modulates the metabolic phenotypic shift of macrophages, contributing to polarization from pro-inflammatory M1 to pro-fibrotic M2 macrophages (39). In a mouse model of PM2.5-induced pulmonary fibrosis, macrophages with increased lactylation secreted more pro-fibrotic factors, which boosted EMT to exacerbate fibrosis through the TGF-β/Smad and VEGFA/ERK pathways. LDHA inhibitor treatment reduced macrophage lactylation and attenuated PM2.5-induced pulmonary fibrosis (78). In conclusion, studying lactylation would enhance our understanding of macrophage mechanisms in pulmonary fibrosis development and lay the groundwork for new immunomodulatory therapeutic strategies.

### 4.3 Lactylation modulates macrophage activation in lung cancer

Macrophage activation largely influences development and progression of lung cancer. Macrophages combat tumors by engulfing and destroying cancer cells, hindering tumor growth and spread (93). Additionally, they release cytokines like interleukins to boost immune cell activation and proliferation (94), bolstering the immune response against tumors. Macrophages also modulate the tumor microenvironment, impacting tumor cell growth, invasion, and metastasis, thereby shaping tumor advancement (95).

Lactate is the end product of glycolysis and a highly abundant metabolite in the tumor microenvironment, and enhanced glycolysis and lactate accumulation are common features of all types of cancer. Tumor cells often rely on glycolytic metabolism for energy production, favoring it over oxidative phosphorylation even in the presence of oxygen. This metabolic preference, known as “aerobic glycolysis” or the “Warburg effect” results in accumulation of large amounts of lactate (96). High levels of lactate in glycolysis-driven tumor cells are linked to increased aggressiveness and poor prognosis, highlighting the role of lactate in cancer progression (97). Indeed, lactate-driven lactacylation promotes lung cancer through a diversity of routes. Defective Numb/Parkin pathway leads to increased histone lactylation and promotes lung adenocarcinoma (LUAD) (98). In lung adenocarcinoma endothelial cells, H3K18la and H3K14la reduce the expression of solute carrier family 25 member 29 (SLC25A29), and low SLC25A29 expression is associated with LUAD progression (99). More importantly, inhibition of histone lactylation could suppress the malignant progression of LUAD (100). Nevertheless, whether and how lactate and lactylation regulate macrophage activation and lung carcinogenesis remain to be investigated.

In lung cancer, M1-type macrophages are primarily activated by cytokines like IL-12, IFN-γ, *etc.* (56). Activated M1-type macrophages induce tumor cell apoptosis and impede tumor growth, thereby suppressing lung cancer development (101). Conversely, activation of M2-type macrophages enhances tumor proliferation, invasion, and metastasis, thereby advancing lung cancer progression (95). Maintaining a balanced M1/M2 macrophage ratio is critical. An unbalanced M1/M2 ratio could alter the microenvironment, fostering tumor growth and metastasis. Twenty-eight lysine lactylation sites on core histones like H3, H4, H2A, and H2B were identified in human HeLa cells and mouse bone marrow-derived macrophages (BMDMs) (13). Studies on oncogenic M2-type macrophages from melanoma and lung cancer tissues in mice have shown a significant positive correlation between lactylation levels and these macrophages, suggesting a potential contribution of elevated lactate and histone lactylation in macrophages to tumor development and malignant advancement. In patients with lung squamous cell carcinoma, solute carrier family 2 member 1 (SLC2A1), encoding glucose transporter-1 (GLUT1), was highly expressed in SPP1^+^ macrophages. Upregulation of SLC2A1 was associated with high levels of protein lactylation and promoted macrophage polarization into the M2 phenotype, which was related to worse survival and poor pathological response to adjuvant immunochemotherapy (102).

## 5 Conclusion and outlook

**Figure 3:**
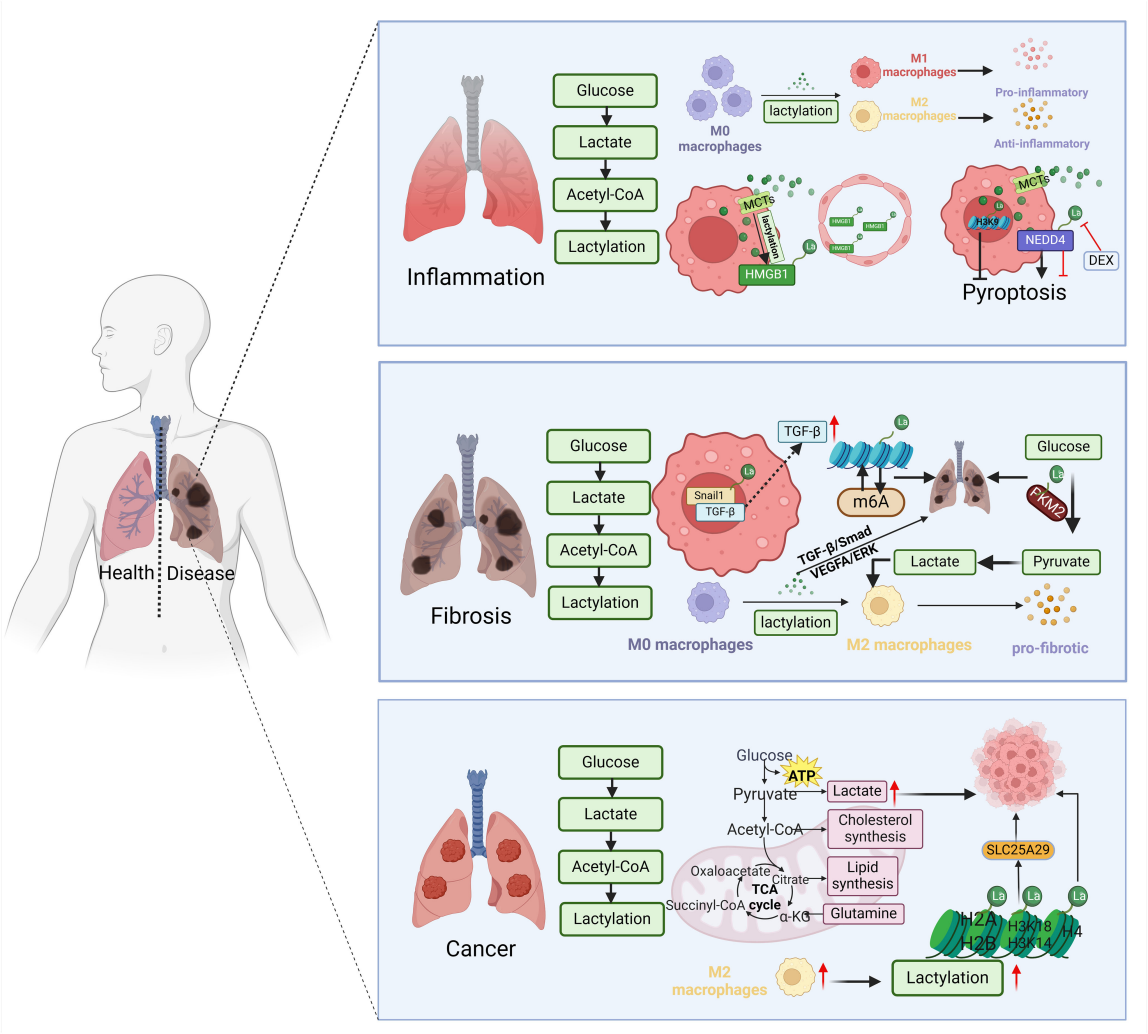
Lactylation modulates macrophage activation in lung disease. During the inflammatory phase, lactylation increases transformation of macrophages into M1 and M2 types. Cellular entry through MCTs boosts HMGB1 lactylation, leading to destruction of endothelial cells. Lactated NEDD4 induces macrophage pyroptosis, while lactated H3K9 inhibits it. Snail1 lactylation at the fibrosis stage stimulates TGF-β secretion, promoting M2 macrophage polarization. Histone lactylation and m^6^A methylation interaction contributes to lung fibrosis. PKM2 methylation enhances macrophage M2 polarization. The glycolysis/gluconeogenesis process in macrophages intensifies during the fibrotic phase. Higher lactate levels are observed in glycolysis-dominant tumor cells. Twenty-eight lysine lactation (Kla) sites were identified on core histones like H3, H4, H2A, and H2B. Oncogenic M2-type macrophages from melanoma and mouse lung cancer tissues exhibit a strong positive association with lactate levels. Figure was created with BioRender.com.

In recent years, there has been a growing focus on the role of lactate as a signaling molecule and lactylation as a post-translational modification of proteins in various physiological and pathological conditions. Understanding how lactate influences macrophage activation is important for gaining insights into disease development. This review highlights key concepts in lactate metabolism, such as the shuttle, homeostasis, and microenvironment interactions of lactate. Recent research has shown that lactate plays a significant role in regulating gene expression related to inflammation, cytokine production, and metabolic pathways in macrophages, ultimately affecting their activation and function. Furthermore, the role of lactylation in modulating macrophage activation in lung diseases like inflammation, fibrosis, and cancer is discussed (**Figure 3**). Abnormal macrophage function is a key factor in the pathogenesis of lung diseases such as pneumonia, pulmonary fibrosis, COPD, and lung cancer, making the study of lactylation particularly relevant in these conditions. Understanding of lactylation’s impact on macrophage activation in lung diseases may offer new opportunities for therapeutic interventions.

Nevertheless, the related research is still in its early stages. Could lactate potentially have similar applications in lung diseases in future? Additionally, could new medications be developed to target lactylation? What is the exact mechanism by which lactate influences macrophage activation, and how does it differ from other pathways of macrophage activation? In conclusion, a thorough exploration of lactylation and its specific role in regulating macrophage activation is essential for advancing our knowledge of lung disease mechanisms, as well as for providing innovative therapeutic approaches and identifying potential targets.

## Author contributions

YW: Writing – original draft. HG: Writing – original draft. SC: Writing – original draft. XT: Writing – original draft, Conceptualization, Funding acquisition, Project administration, Resources, Supervision, Writing – review & editing.
